# Characterization of the blood–brain barrier in genetically diverse laboratory mouse strains

**DOI:** 10.1186/s12987-021-00269-w

**Published:** 2021-07-28

**Authors:** Johanna Schaffenrath, Sheng-Fu Huang, Tania Wyss, Mauro Delorenzi, Annika Keller

**Affiliations:** 1 Department of Neurosurgery, Clinical Neuroscience Center, University Hospital Zürich, Zürich University, Zürich, Switzerland; 2 Neuroscience Center Zürich, University of Zürich and ETH Zürich, Zürich, Switzerland; 3 Bioinformatics Core Facility, Swiss Institute of Bioinformatics, Lausanne, Switzerland; 4 Department of Oncology, University Lausanne, Lausanne, Switzerland

**Keywords:** Blood–brain barrier, Brain endothelial cells, Vascular zonation, Vascular permeability, Inbred mouse strains

## Abstract

**Background:**

Genetic variation in a population has an influence on the manifestation of monogenic as well as multifactorial disorders, with the underlying genetic contribution dependent on several interacting variants. Common laboratory mouse strains used for modelling human disease lack the genetic variability of the human population. Therefore, outcomes of rodent studies show limited relevance to human disease. The functionality of brain vasculature is an important modifier of brain diseases. Importantly, the restrictive interface between blood and brain—the blood–brain barrier (BBB) serves as a major obstacle for the drug delivery into the central nervous system (CNS). Using genetically diverse mouse strains, we aimed to investigate the phenotypic and transcriptomic variation of the healthy BBB in different inbred mouse strains.

**Methods:**

We investigated the heterogeneity of brain vasculature in recently wild-derived mouse strains (CAST/EiJ, WSB/EiJ, PWK/PhJ) and long-inbred mouse strains (129S1/SvImJ, A/J, C57BL/6J, DBA/2J, NOD/ShiLtJ) using different phenotypic arms. We used immunohistochemistry and confocal laser microscopy followed by quantitative image analysis to determine vascular density and pericyte coverage in two brain regions—cortex and hippocampus. Using a low molecular weight fluorescence tracer, sodium fluorescein and spectrophotometry analysis, we assessed BBB permeability in young and aged mice of selected strains. For further phenotypic characterization of endothelial cells in inbred mouse strains, we performed bulk RNA sequencing of sorted endothelial cells isolated from cortex and hippocampus.

**Results:**

Cortical vessel density and pericyte coverage did not differ among the investigated strains, except in the cortex, where PWK/PhJ showed lower vessel density compared to NOD/ShiLtJ, and a higher pericyte coverage than DBA/2J. The vascular density in the hippocampus differed among analyzed strains but not the pericyte coverage. The staining patterns of endothelial arteriovenous zonation markers were similar in different strains. BBB permeability to a small fluorescent tracer, sodium fluorescein, was also similar in different strains, except in the hippocampus where the CAST/EiJ showed higher permeability than NOD/ShiLtJ. Transcriptomic analysis of endothelial cells revealed that sex of the animal was a major determinant of gene expression differences. In addition, the expression level of several genes implicated in endothelial function and BBB biology differed between wild-derived and long-inbred mouse strains. In aged mice of three investigated strains (DBA/2J, A/J, C57BL/6J) vascular density and pericyte coverage did not change—expect for DBA/2J, whereas vascular permeability to sodium fluorescein increased in all three strains.

**Conclusions:**

Our analysis shows that although there were no major differences in parenchymal vascular morphology and paracellular BBB permeability for small molecular weight tracer between investigated mouse strains or sexes, transcriptomic differences of brain endothelial cells point to variation in gene expression of the intact BBB. These baseline variances might be confounding factors in pathological conditions that may lead to a differential functional outcome dependent on the sex or genetic polymorphism.

**Supplementary Information:**

The online version contains supplementary material available at 10.1186/s12987-021-00269-w.

## Background

Dysfunction of brain vasculature and the blood–brain barrier (BBB) has emerged as an important comorbidity and modifier of brain diseases. Furthermore, increased BBB permeability has been reported to occur during aging [1, 2]. A better understanding of BBB and vascular changes during aging and in brain diseases is essential for the development of vasoprotective therapies, and for facilitating drug delivery into diseased brain. A reductionist approach using loss-of-function mouse models in a single genetic background has been successful in identifying genes important for BBB development [3]. However, this approach will most likely be ineffective for identifying genes and pathways in humans that influence BBB alterations during aging and in brain diseases. Mouse studies have shown that genotype–phenotype relationships cannot be reliably inferred by studying a single genetic background [4] because inbred strains fail to mimic the genetic and physiological complexity in humans. The genetic variability of each individual can have profound effects on the presentation of even monogenic diseases [5]. The identification of genetic modifiers in humans is very difficult due to differences in environmental factors and the vast genetic variability in the natural population. Furthermore, studies on human brain vasculature (and brain in general) are hindered due to the scarcity of tissue available for such analyses. Most studies on BBB development as well as alterations of the BBB in aging and disease (e.g. Alzheimer’s, ischemic stroke) are carried out in a C57BL/6J background, the strain with the lowest number of single nucleotide polymorphisms (SNPs) [6]. However, this limited genetic variability can be overcome by applying a systems biology approach and investigating several inbred mouse lines and reference populations. This strategy provides a controlled approach to study variation that influences a certain phenotype. Experimental models of cardiovascular disease or adaptation to exercise largely depend on genetic background and differences in endothelial function (e.g. ACh dose–response curves) [7, 8]. In the CNS, inbred mouse strains show differences in neurogenesis [9–11], in behavioral performance [12], ischemic stroke volume [13], regenerative capacities [14], and cerebral collateral vessel density [15]. Furthermore, the genetic background of mice influences the deposition of amyloid and myeloid-driven neuroinflammation in murine models of Alzheimer’s disease [16].

The BBB is a dynamic interface that actively responds to changes in neural tissue and metabolism. The genetic background of mouse models may not only affect adaptive changes but also severity of BBB alterations during aging and brain diseases. Characterization of the phenotypic heterogeneity of the BBB in genetically distinct mouse strains may yield insights into how genetic variation influences the BBB characteristics.

In this study, we investigated the phenotypic heterogeneity of vasculature and endothelial cells (EC) using several inbred mouse strains: 129S1/SvlmJ, DBA/2J, A/J, C57BL6J, NOD/ShiltJ, WSB/EiJ, PWK/PhJ and CAST/EiJ. WSB/EiJ, PWK/PhJ and CAST/EiJ are recently wild-derived (1980-s) and present highest genetic variation whereas 129S1/SvlmJ, DBA/2J, A/J, C57BL6J, NOD/ShiltJ strains present very limited intra-strain polymorphism [6, 17, 18]. We characterized brain vasculature using three phenotypic arms—analysis of vascular density, arteriovenous (A-V) zonation and pericyte coverage, BBB permeability to sodium fluorescein, and transcriptomic analysis of EC using RNA sequencing. Investigated inbred strains showed similar vessel density and pericyte coverage, endothelial zonation pattern, and permeability to sodium fluorescein in the cortex and hippocampus. Transcriptomic analysis of brain EC revealed sex dimorphism in genes involved in mitochondrial function. Transcriptome of EC from recently wild-derived and long-inbred strains revealed differential expression of genes involved in vascular development and homeostasis, immune system response, and membrane transport. To summarize, we describe the spectrum of variation in intact BBB and brain vasculature in healthy young mice of different strains as well as age-related changes in some long-inbred strains.

## Results

### Analysis of vessel density and pericyte coverage in inbred mouse strains

**Fig. 1:**
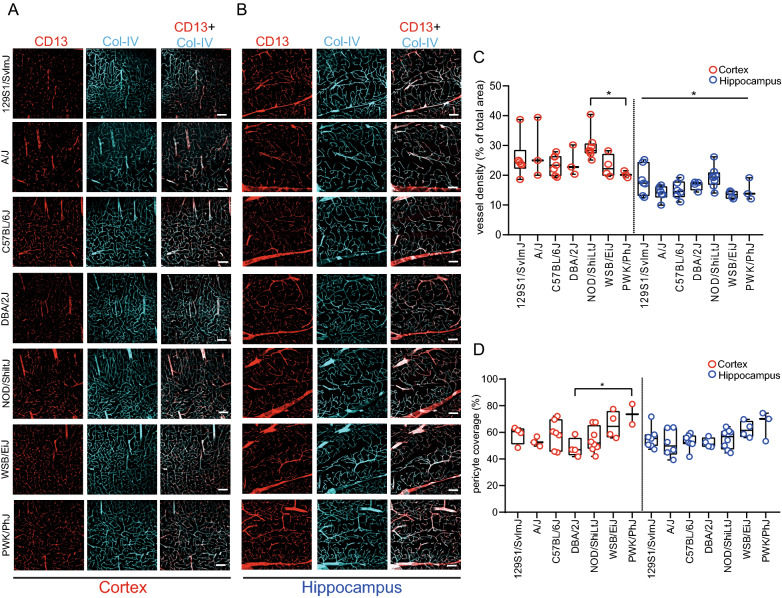
Vessel density and pericyte coverage are similar between different inbred strains. Immunofluorescence staining of CD13 (in red, pericyte marker) and vascular basement membrane (Col-IV, in cyan) in cortex (**A**) and hippocampus (**B**). Quantification of vessel density (**C**) and pericyte coverage (**D**) in cortex (red) and hippocampus (blue). Each dot indicates one mouse (**C** and **D**). N = 3–8. Female and male mice were used for analysis. Age of mice: 2 months. Statistical testing performed using Kruskal–Wallis with Dunn`s multiple comparison test (vessel density in cortex) or one-way ANOVA with Tukey`s multiple comparison test (vessel density in hippocampus, **D**). *—p < 0.05. Data are presented as mean ± SD. Scale bars: 100 µm

We carried out vessel density measurement in the cortex and hippocampus of well-characterized, common laboratory strains: 129S1/SvlmJ, DBA/2J, A/J, C57BL6J, NOD/ShiltJ, and recently wild-derived strains: WSB/EiJ and PWK/PhJ. Vasculature was visualized using the basement membrane protein collagen-IV (Fig. 1a, b). There were no detectable differences in vessel density in the cortex of all investigated stains, except for NOD/ShiLtJ. (Fig. 1c). The Kruskal–Wallis test and post-hoc analysis using Dunn`s test showed that NOD/ShiLtJ mice had increased vessel density in the cortex compared to PWK/PhJ (Fig. 1c). The overall variance in vessel density differed in the hippocampus (One-way ANOVA, Fig. 1c). Pericytes are implicated in the regulation of BBB permeability [19–21]. Therefore, we assessed the pericyte coverage of brain vasculature in the cortex and hippocampus. Pericytes were visualized using anti-CD13 antibody (Fig. 1a, b). Wild-derived strain PWK/PhJ had a significantly higher pericyte coverage in the cortex compared to DBA/2J (One-way ANOVA, post-hoc Tukey test, Fig. 1d). In the hippocampus, pericyte coverage did not differ between analyzed strains (Fig. 1d). In conclusion, our analysis shows slight differences in vessel density and pericyte coverage in the cortex and hippocampus among analyzed mouse strains.

### Analysis of endothelial zonation in inbred mouse strains

**Fig. 2:**
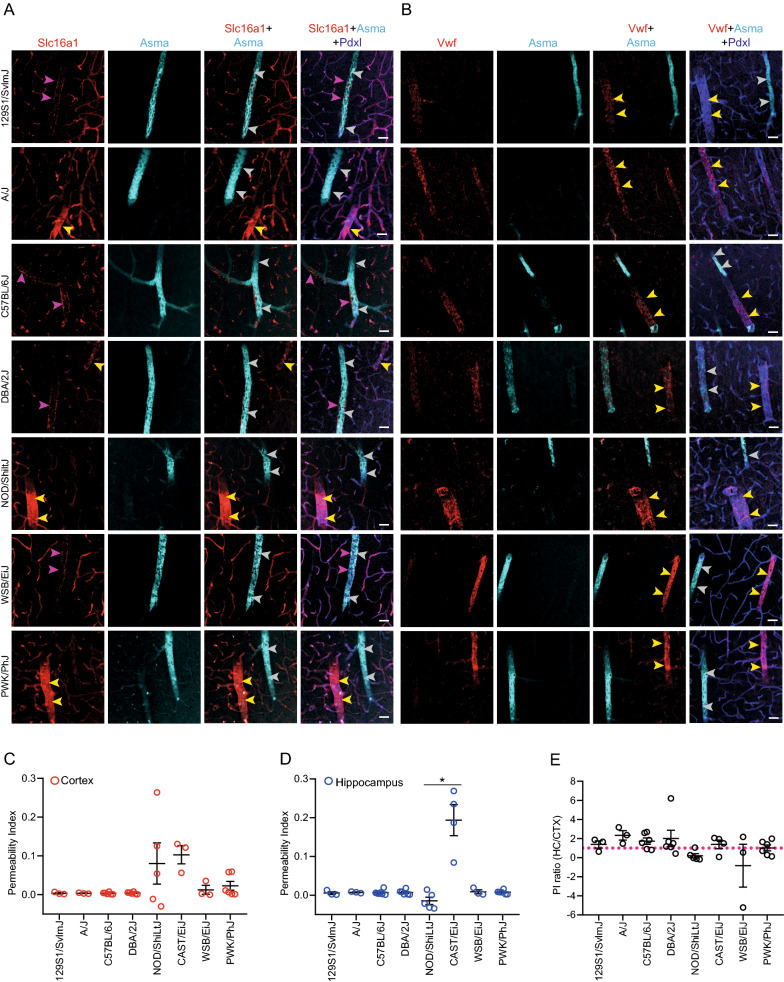
Vascular zonation and BBB permeability are similar between different mouse strains. Immunofluorescence staining of Slc16a1 (venous and capillary EC marker, in red) (**A**), Vwf (venous marker, in red) (**B**) Asma (arterial VSMC marker, in cyan) and podocalyxin (Pdxl, endothelial lumenal marker, in blue) (**A** and **B**) in different inbred strains. Grey arrowheads point to arteries and yellow arrowheads to veins. Permeability Index (PI) to NaF of cortex (**C**) and hippocampus (**D**) in different mouse strains. The PI ratio (hippocampus/cortex) of NaF in different mouse strains (**E**). Pink dotted line: PI ratio = 1. Each dot indicates one mouse (**C-E**). N = 3–6. Female and male mice were used for analysis. Age of mice: 2 months. Data are presented as mean ± SD. PI—permeability index; HC—hippocampus; CTX—cortex. Statistical testing was performed using Kruskal–Wallis with Dunn`s multiple comparison test.*—p < 0.05. Scale bars: 30 µm

We next asked whether different mouse strains exhibit a similar endothelial A-V zonation pattern as recently described in C57BL/6J animals [22]. In order to assess endothelial zonation, we used a set of markers, which are differentially expressed depending on the identity of EC on the vascular tree. Slc16a1 (monocarboxylate transporter, MCT1) is strongly expressed by venous and capillary endothelium, whereas Vwf is expressed by venous and arterial endothelium and not by capillary endothelium [22]. Immunofluorescence of Slc16a1 and Vwf in combination with podocalyxin and smooth-muscle actin in different strains did not show differences in staining patterns between strains (Fig. 2a, b). In all analyzed strains, Slc16a1 was expressed by capillaries and venous endothelium (Fig. 2a), whereas Vwf was expressed strongest by venous endothelium (Fig. 2b). Slc16a1 positivity was also detected outside the vascular smooth cell layer (Fig. 2a, pink arrowheads), which could represent astrocyte end-feet. Thus, based on analyzed markers, the endothelial A-V zonation did not differ between investigated strains and was consistent with previously published endothelial A-V zonation patterns in the C57BL/6J strain [22].

### Analysis of BBB permeability to sodium fluorescein in inbred mouse strains

We next assessed BBB permeability to the intravenously administered small molecular weight tracer sodium fluorescein (NaF, 376 Da) in the cerebral cortex and hippocampus. Increased permeability to NaF is thought to indicate subtle changes in paracellular BBB permeability [23]. Permeability to NaF (*Permeability Index—PI*) was calculated using the previously published approach [24]. The BBB of young, healthy mice is not expected to show high vascular permeability. Accordingly, the PI of NaF is low in investigated strains (Fig. 2c, d). We observed among tested mouse strains that the CAST/EiJ mice showed the highest PI in the cortex and hippocampus (Fig. 2c, d), and a statistically significant difference in PI among NOD/ShiLtJ and CAST/EiJ strains in the hippocampus (Fig. 2d). Neurogenic region in the lateral ventricle has been shown to have a higher permeability to NaF in C57BL6/J animals [25]. Overall, we observed equal permeability to NaF in hippocampi compared to cortices of different strains (Fig. 2e). Only A/J mice showed higher PI (2.3) in hippocampus compared to cortex and NOD/ShiLtJ mice showed lower PI (0.19) in hippocampus compared to cortex (Fig. 2e). Thus, there were no major overall differences in the BBB permeability to NaF in all analyzed strains.

### Age related changes in vascular morphology and BBB function

**Fig. 3:**
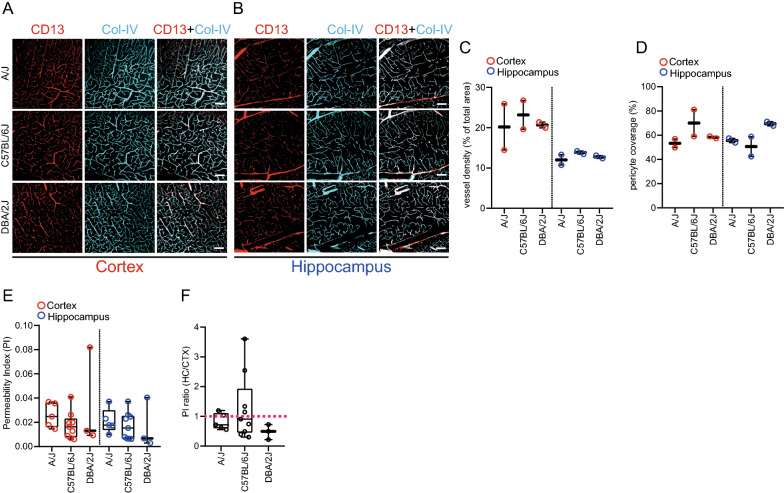
Vessel density, pericyte coverage and permeability index to NaF of 12 month old mice. Immunofluorescence staining of CD13 (in red, pericyte marker) and vascular basement membrane (Col-IV, in cyan) in the cortex (**A**) and hippocampus (**B**). Quantification of vessel density (**C**) and pericyte coverage (**D**) in the cortex and hippocampus. N = 2. PI of cortex or hippocampus (**E**) and ratio of PI hippocampus to PI cortex (**F**) of different mouse strains of 12 months old animals. N = 3–9. Pink dotted line: PI ratio = 1. Each dot indicates one mouse (**C**–**F**). Age of mice: 12 months. Female and male mice were used for analysis. Statistical testing performed using ANOVA. Data are presented as mean ± SD. Scale bars: 100 µm

We next assessed vascular and BBB permeability changes during aging. We chose three mouse strains (A/J, C57BL/6J, DBA/2J) with a different mean lifespan (Additional file 1: Fig. S1a) [26] in order to investigate differences in BBB permeability alteration during aging [1, 27, 28]. First, we quantified vessel density and pericyte coverage in 12 months old mice and compared these parameters with 2 month old mice. No significant difference in vessel density or pericyte coverage was observed between investigated aged strains (Fig. 3a–d). Cortical vessel density and pericyte coverage did not significantly differ between young and aged animals within strains (Additional file 1: Fig. S1b, c). Similar observations were made in the hippocampus for all investigated strains, expect DBA/2J. Aged DBA/2J mice showed significantly lower vessel density and significantly higher pericyte coverage compared to 2 months old DBA/2J mice in the hippocampus (Additional file 1: Fig. S1b, c).

We next analyzed vascular permeability to NaF in the aged mice. No significant changes in the PI of the cortex and hippocampus of 12-month-old animals were observed among analyzed strains (Fig. 3e). Also, there were no significant changes in the ratio of hippocampal/cortical PI among the strains (Fig. 3f). A comparison of cortical PI between 2- and 12-month old animals showed an increase of PI in older animals (Additional file 1: Fig. S1d). Similarly, there was an increase in hippocampal PI between 2 and 12 month old animals in all investigated strains (Additional file 1: Fig. S1e). Thus, there is a modest increase in BBB permeability to NaF in the cortex and hippocampus in aged mice of different inbred strains except for A/J animals where a comparison of the ratio of hippocampal and cortical PI between 12 and 2 month old animals showed a decrease (Additional file 1: Fig. S1f).

### Analysis of the brain endothelial cell transcriptome in inbred mouse strains

**Fig. 4:**
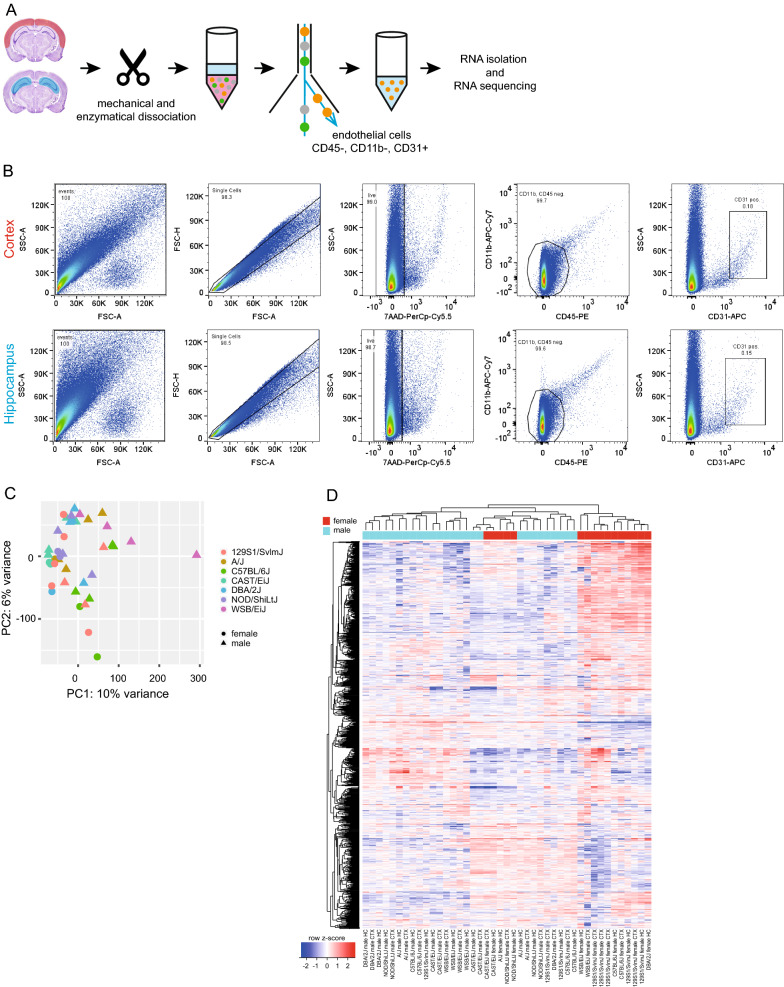
RNA sequencing of cortical and hippocampal endothelial cells of different inbred mouse strains. Brain endothelial cells for RNA sequencing were isolated from dissected hippocampus and cortex after mechanical and enzymatical dissociation. Age of mice: 2 months. Isolation of EC using anti-CD31 Ab labelling and flow cytometry (**A**). Pseudocolor flow cytometry dotplots showing the gating strategy to sort for CD31^+^ endothelial cells. Gating included singlet gating, live cell gating (7AAD^neg^), exclusion of immune cells (CD11b^+^, CD45^+^) and inclusion of CD31^+^ endothelial cells (**B**). Principal component analysis of normalized RNAseq counts of the deregulated genes (**C**). Hierarchical clustering heatmap showing z-scores of normalized read counts for the 1000 most variable genes across samples (**D**)

In order to characterize the transcriptome of brain EC of different mouse strains, we isolated EC originating from the entire vascular tree (i.e., arterioles, capillaries, venules) from the cortex and hippocampus using fluorescent activated cell sorting (FACS) (Fig. 4a, b). Manual gating of CD31^+^ EC is shown in Fig. 4b. Using a purity mask, EC were sorted directly into lysis buffer for RNA extraction and subsequent RNA sequencing.

Raw sequencing reads were aligned to the *Mus Musculus* genome (mm10) and raw counts were summarized per gene for each sample. Data analysis was performed using the online platform iDEP.92 [29]. Samples with good coverage were used for further analysis. We used gene set enrichment analysis to determine whether our dataset was enriched for endothelial core genes [22]. A high positive average expression values for EC genes showed that we had successfully enriched for EC (Additional file 1: Fig. S2a). Vascular cell contamination is a common problem when analyzing endothelial cell transcriptional profile because vascular cells are difficult to separate. High average expression values for vascular smooth muscle cell, pericyte, and astrocyte genes in our dataset indicated that although we had enriched for EC, we had not achieved a pure endothelial cell population (Additional file 1: Fig. S2b–d). However, expression values of mural and astrocyte genes were lower compared to EC genes. Interestingly, no enrichment was observed for microglia or vessel-associated fibroblast genes (Additional file 1: Fig. S2e, f). Principal component analysis (PCA) showed PC1 explaining 10% of variation tended to separate samples according to the sex—male *vs* female (Fig. 4c). Unsupervised hierarchical clustering of the dataset based on the 1000 most differently expressed genes generated three main clusters: one consisting of only male samples (blue bar), another consisting of only female samples (red bar) and the third cluster consisting of both male and female samples (Fig. 4d, Additional file 2: Table S1).

**Fig. 5:**
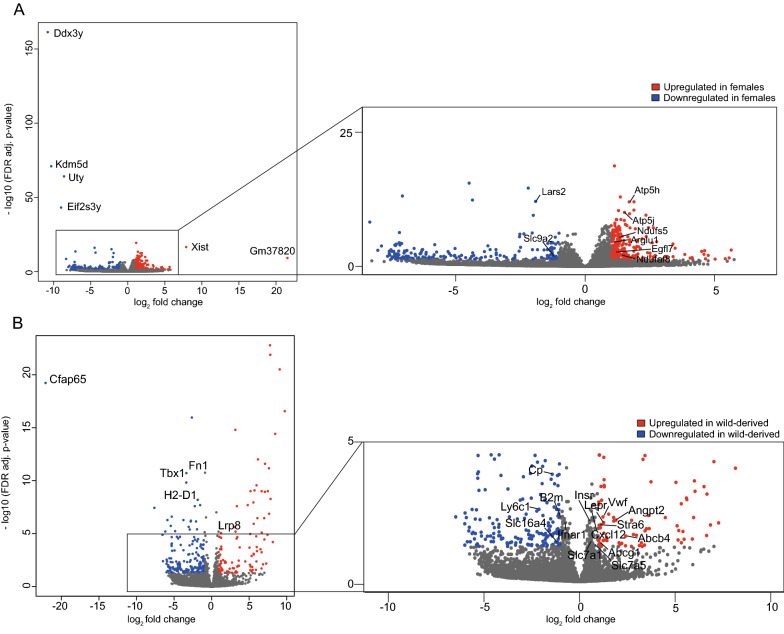
Differently expressed genes (DEG) of CD31^+^ brain endothelial cells isolated from brains of different inbred mouse lines. Volcano plot showing DEG (FDR value cutoff 0.05, min fold change of 2) for comparing samples based on sex (female *vs* male) (**A**) or genetic variability (recently wild-derived *vs* long-inbred strains) (**B**)

First, we investigated sex-dependent transcriptomic changes in brain EC. Differentially expressed gene (DEG) analysis showed that 947 genes were deregulated when comparing female *vs* male samples (false discovery rate (FDR) value < 0.05) (Fig. 5a, Additional file 3: Table S2). Three hundred and eighty eight genes were down- and 609 genes were upregulated in female EC compared to male EC. In our dataset, we detected several female sex-specific (e.g. *Xist*, *Arglu1*) and male sex-specific genes (e.g. *Ddx3y*, *Kdm5d, Uty)* (Fig. 5a). Gene Ontology (GO) overrepresentation analysis of DEGs (FDR < 0.05) revealed mRNA processing, mitochondrial function and translation associated genes within the significantly upregulated gene sets in female EC (Additional file 3: Table S2). We found a significantly higher expression of components of mitochondrial ATP synthase (e.g. *Atp5h, Atp5j*) and respiratory complex 1 (e.g. *Ndufs5, Ndufaf8*) in female EC compared to male EC (Fig. 5a, Additional file 1: Fig. S3a). In addition to differential expression of female and male sex-specific genes, and genes encoding mitochondrial proteins, we detected differential expression of several genes predominantly (e.g. *Egfl7) *[22] or only (*Slc9a2*) expressed in brain EC (Fig. 5a, Additional file 1: Fig. S3b). Interestingly, we also detected differential expression of *Lars2*, a gene encoding mitochondrial leucyl-tRNA synthetase 2 (Fig. 5a, Additional file 1: Fig. S3b), previously suggested to be differentially expressed by male and female EC [30].

We next compared the endothelial cell transcriptome of recently wild-derived strains (CAST/EiJ, WSB/EiJ), containing the highest number of SNPs [6], with those bred in a laboratory for several decades and presenting limited inter-strain polymorphism (long-inbred strains—A/J, DBA/2 J, NOD/ShiLtJ, 129S1/SvImJ and C57BL/6J) [6]. DEG analysis showed that 395 genes were deregulated (227 genes were down- and 168 genes were upregulated) when comparing recently wild-derived strains samples to long-inbred strains (FDR value < 0.05) (Fig. 5b, Additional file 4: Table S3). Interestingly, many DEGs were either processed (*Gm13394, Gm10709*) or unprocessed pseudogenes (*Gm13339, GM13340*). Deregulated pseudogenes were ribosomal protein pseudogenes (e.g. *Rps12-ps10*, *Gm10709* (L29), *Gm8172* (S6)) or encoding for noncoding RNAs—lncRNAs (e.g. *Gm46218, Gm30075*), ncRNA (e.g. *Gm10069, Gm35572*), snRNA (e.g. *Gm25939, Gm22513*) (Additional file 4: Table S3).

Endothelial transport is critical for the brain function. We identified differential expression of genes encoding for transporters between recently wild-derived and long-inbred strains. Expression of *Slc16a4* (MCT5), a monocarboxylate transporter, was downregulated, whereas expression of amino acid transporters *Slc7a1* and *Slc7a5*, were upregulated in EC of wild-derived mice compared to long-inbred mice (Fig. 5b, Additional file 1: Fig. S4a, Additional file 4: Table S3). Wild-derived mice showed higher expression of several ABC transporters involved in lipid transport—*Abcg1* (a sterol transporter controlling intracellular sterol levels), *Abcb4* (a phosphatidylcholine transporter) and *Stra6 (*a retinol transporter) (Fig. 5b, Additional file 1: Fig. S4b, Additional file 4: Table S3). In addition, we detected deregulated expression of genes involved in receptor-mediated transport at the BBB. EC of recently wild-derived mice expressed higher levels of the *Insr (*insulin receptor*)*, and *Lepr* (leptin receptor) genes (Fig. 5b, Additional file 1: Fig. S4c, Additional file 4: Table S3).

In addition to endothelial transporters, we identified differential expression of several genes, implicated in vascular development and homeostasis. For example, recently wild-derived mice expressed higher levels of *Vwf*, *Lpr8*, *Angpt2*, *Cxcl12* and lower levels of *Fn1* (basement membrane protein) and *Tbx1* (transcription factor) (Fig. 5b, Additional file 1: Fig. S4d, e, Additional file 3: Table S3). Wild-derived mice compared to long inbred mice showed differences in susceptibility to viral and bacterial infections, and immune response [31, 32]. Accordingly, we detected also differences in EC expression of genes that play a role in immune response—*Ly6c1*, *H2-D1, B2m*, *Cp* or *Ifnar1* (Fig. 5b, Additional file 1: Fig. S4f, Additional file 4: Table S3).

In summary, the main variation of EC transcriptome isolated from different inbred strains was due to the sex of the animal. However, comparison of EC transcriptomes between recently wild-derived and long inbred strains revealed DEGs, suggesting differences in endothelial transport and immune response.

## Discussion

In this study, we characterized the BBB of eight inbred mouse strains; A/J, C57BL/6 J, CAST/EiJ, DBA/2J, NOD/ShiLtJ, PWK/PhJ, WSB/EiJ, and 129S1/SvImJ using several phenotypic arms. We quantified vessel density, pericyte coverage, BBB permeability to NaF, and characterized endothelial A-V zonation pattern. In addition, we performed genome-wide transcriptional profiling of brain EC.

Inbred mouse strains differ in collateral vessel density [15]; however, less is known regarding the extent of differences of brain vascular topology between inbred mouse strains. Recent methodological advances have already yielded brain-wide imaging of vasculature of different strains (C57BL/6J, CD1 and BALB/c) [33]. Our study complements and extends previous investigations. Although not brain-wide, we provide a high-resolution characterization of A-V zonation pattern, vessel density and pericyte coverage in different young inbred mouse strains in two brain regions. The analyzed strains showed variable vessel density in the hippocampus. Interestingly, wild-derived PWK/PhJ showed decreased vessel density in the cortex compared to NOD/ShiLtJ and greater pericyte coverage in the cortex than DBA/2J. Analysis of vessel density and pericyte coverage in aged (12 month old) A/J, C57BL6/J and DBA/2J mice, which have a different life span, showed reduced vascular density in the hippocampus in old DBA/2J mice. Previous studies have shown that vessel density and pericyte coverage decreases during ageing in C57BL6/J mice, both in the brain and in peripheral organs with low regenerative capacity [34, 35]. We did not detect reduced vascular density in brains of C57BL6/J mice. However, our analysis was performed in relatively thin (22 µm) z-stack confocal images in two brain regions. Although our images for quantification of vascular parameters were of high resolution, it is likely that we did not reach high enough volumes to detect changes in vessel density and pericyte coverage.

We investigated whether different mouse strains present different baseline permeability to NaF, a small molecular weight tracer. As expected, we did not detect statistically significant differences in permeability to NaF among the investigated mouse strains. However, NOD/ShiLtJ and CAST/EiJ mice showed higher PI in the cortex compared to other strains. Previous studies in C57BL6/J animals have shown a higher vascular permeability to NaF in the neurogenic region along the lateral wall of the lateral ventricle [25]. Except for A/J, our analysis of BBB permeability did not show an elevated PI index in the hippocampus in C57BL6/J animals and other investigated strains. Thus, if neurogenic zones in a mouse brain have a higher BBB permeability compared to other regions, it might be restricted to a certain zone and/or this characteristic is strain specific. Prolonged isoflurane anesthesia in old C57BL/6J female mice was shown to influence the BBB permeability [36]. CAST/EiJ were anesthetized with isoflurane before tail vein injection, which could potentially increase BBB permeability. However, PWK/PhJ and WSB/EiJ strains, which show a similar PI as non-anesthetized strains (e.g. C57BL/6J), were also anesthetized before tail vein injection. Therefore, if isoflurane anesthesia had caused an increase in BBB permeability, this would indicate a strain specific effect. In healthy mice, the brain penetration mode of NaF has been described to be passive [37], however, NaF is a general substrate for transporters of the SLCO family and MRP2 (*ABCC2*) [38, 39]. Several SLCO transporters are expressed by mouse brain EC [22] and SLCO2B1 is expressed by human brain endothelium [40]. Interestingly, streptozotocin-induced diabetes led to decreased vascular permeability to fluorescein paralleled by increased expression of Mrp2 in rat brain [41], a transporter that has been shown to be differentially expressed in cerebral blood vessels in C57BL/6J and FVB strains [42]. However, our transcriptional analysis did not identify any differences in expression levels of transcripts encoding for Slco1c1, Slco2b1 and Mrp2 within analyzed strains. In accordance with published literature [2, 27], we detected an increase in BBB permeability during aging, both in the hippocampus and cortex. BBB permeability changes in cortex within three investigated strains showed a different slope. It would be interesting to investigate whether the observed differences are due to the genetic background of animals.

The investigated mouse strains did not show major differences in BBB permeability to NaF in young mice. However, due to differences in the genetic background, we assumed that the transcriptome of EC isolated from different stains would show gene expression changes reflecting normal variation of the transcriptome BBB. Unexpectedly, the largest variation in the obtained RNAseq dataset was not due to the genetic background of mice but sex. Interestingly, we found that EC isolated from female mice express higher levels of genes associated with mitochondrial function. Female and male cells show differences in mitochondrial function (e.g. oxygen consumption, ATP production) in various tissues in both rodents and human [43–45]. In addition, analyses of genetically diverse mice have shown that mitochondrial function is also dependent on genetic background [46]. Mitochondrial dysfunction is associated with diseases, including cardiovascular diseases and neurodegeneration. Since vascular dysfunction modifies brain diseases, future studies should investigate the impact of mitochondrial dysfunction due to sex differences and its potential impact on pathology and tissue repair. A recent study by Paik et al. [30] identified higher expression of *Ddx3y, Zbtb16, Gm6981, Mapk6* genes in EC of male brain and *Xist, Tsix, Arglu1, Twf1 and Plp1* genes in EC of female brain [30]. The authors concluded the existence of brain EC-specific sex-dependent genes. However, many of the identified genes are encoded by X- and Y- chromosomes and used as markers for sex determination (e.g. *Ddx3y*, *Xist*). Thus, these findings cannot represent a true organ-specific EC difference. Similar to other studies [30, 47], we identified sexually dimorphic expression of *Lars2,* a gene encoding a leucyl-tRNA synthase 2 catalyzing the ligation of leucine to its cognate tRNAs. Mutations in *LARS2* cause Perraut syndrome, a rare autosomal recessive disorder causing sensory sensorineural hearing loss in both genders and ovarioleukodystrophy in females [48]. It remains to be determined whether Lars2 affects mitochondrial function in a gender dependent manner and whether dysfunction of brain endothelial cells contributes to the ovarioleukodystrophy. A recent proteomic study of rat brain microvessels also identified sexual dimorphism with females expressing more mitochondrial proteins involved in energy production including the OXPHOS pathway [49].

We compared the EC transcriptome of recently wild-derived with long-inbred strains, which show low inter-strain polymorphism. We identified differential expression of several EC-specific genes. Our results corroborated previous findings that Vwf expression differs between inbred strains (e.g. lower levels in C57BL/6 J mice compared to WSB/EiJ mice) [50]. Vwf is a key protein for vascular homeostasis and angiogenesis. In addition, several genes implicated in the formation of vasculature or regulating endothelial responses in different pathological settings (*Lrp8, Angpt2, Cxcl12*) [51–53] are differently expressed in inbred strains. We found that a brain endothelial specific gene *Lrp8* (Apoer2) is upregulated in EC of recently wild-derived mice. Apoer2 mediating Reelin signaling in EC is necessary for cerebral cortex vascularization and BBB maturation [53]. Genetic variation in the LRP8 gene in humans was shown to influence myocardial infarction and the early onset of coronary artery disease [54]. Interestingly, the binding of ApoE3 to ApoER2 stimulates endothelial NO synthase (eNOS) [55]. It would be interesting to investigate whether differences in the expression of *Vwf, Lrp8* genes and transporters/receptors (e.g. *Slc7a5, Insr, Lepr, Stra6*), and immune response genes (e.g. *B2m*, *Ifnar1, H2-D)* in EC have functional consequences in the brains of adult mice during aging or pathological process. For example, whereas the contribution of Stra6-mediated transport to total retinol uptake by tissues other than the eye is rather modest, Stra6 has been shown to induce endothelial inflammation via circulating RBP [56, 57]. Interestingly, several ATP-dependent efflux pumps, which could affect bioavailability of drugs in the CNS, show a high polymorphism in human population that affects expression level and function [58]. Also, our analysis has identified differential expression of transcripts encoding ABC transporters (Abcg1, Abcg4, Abcg3) in wild-derived and long-inbred strains. Further studies are needed to determine whether changes in mRNA level alter protein level and activity.

In summary, we provide a detailed description of BBB variation in healthy young and aged mice of different inbred strains. Further analysis of complex BBB trait in inbred mice during aging and pathological conditions is likely to yield insight on the influence of sex and genetic diversity on the BBB response during different metabolic conditions (e.g. fasting, high-fat diet), response to injury, and repair processes in mice. Future challenges include the development of methods that will allow quantitative assessment of individual BBB properties (e.g. transport properties, EC junctional organization, inflammatory state etc.) and linking these characteristics with various functional outcomes (e.g. blood flow, neuronal activity).

## Materials and methods

### Mice

Mice were bred in house and pups were separated at postnatal day 21. The following mouse strains were used for the experiments—A/J, 129S1/SvlmJ, C57BL/6J, NOD/ShiLtJ, PWK/PhJ, WSB/EiJ, CAST/EiJ, DBA/2J. Age of mice was 8 weeks ± 2 weeks or 12 months ± 3 weeks. Both, male and female mice were used. All mice were housed in Type 2L cages (530 cm^2^, max 5 mice per cage) with individual ventilation under specific-pathogen-free conditions and a 12 h light/dark cycle. Cage environment included a red polycarbonate house and shredded paper nesting material. Water and food (cat #3336, #3436 KLIBA NAFAG) were provided ad libitum. All mice were housed in the same animal house, in the same room. Experimental procedures were approved by the Cantonal Veterinary Office Zurich (ZH151/2017).

### Analysis of BBB integrity using sodium fluorescein

Sodium fluorescein (NaF, Sigma F6377) was diluted in 0.9% NaCl (8 mg/ml) and 1.2 mg was injected into the tail vein. The tracer circulated for 2 h. Before restraining and tail vein injection, CAST/EiJ, WSB/EiJ and PWK/PhJ mice were anesthetized with isoflurane using an anesthesia system with a vaporizer and chamber (VetEquip.Inc) for 3–4 min because of difficulties in capturing and holding recently wild-derived strains [59]. However, mice were conscious during the tracer circulation time. After 2 h, animals were deeply anesthetized using Ketamine (200 mg/kg) and Xylazine (20 mg/kg). After a loss of response to reflex stimulation (toe-pinch), they were transcardially perfused with PBS. Prior to perfusion, cardiac blood was withdrawn and serum was collected for further analysis using a BD microtainer (BD, #365,968). After perfusion with PBS, brains were removed and hippocampi and cerebral cortex were dissected. Dissected tissue was weighed and lysed in 1% Triton X-100 in PBS using a Qiagen TissueLyser LT (2 × 5 min, 50 osc/s) and centrifuged for 10 min at RT, 13,000 rpm. Supernatants were collected for further analysis. Tissue supernatants and serum were analyzed in 96 well plates (Thermo Scientific, 265,301) using a spectrophotometer from Spectramax Paradigm. Samples were excited using wavelength of 492 nm and signal was collected in a bottom read mode in 2.5 mm reading height an emission wavelength of 525 nm (Software-Soft Max Pro 6.2.). Brain permeability to NaF was calculated using a formula (modified from Devraj et al. [24]).

$$PI=\frac{{tissue (g)fluorescence}_{injected}- \underline{x} ({tissue (g)fluorescence}_{uninjected})}{{serum \left(mL\right)fluorescence}_{injected}-\underline{x} ({serum \left(mL\right)fluorescence}_{uninjected})}$$

Data analysis was performed in Microsoft Excel and GraphPad Prism 8.

### Immunohistochemistry

Mice were deeply anesthetized using Ketamine (200 mg/kg) and Xylazine (20 mg/kg) after a loss of response to reflex stimulation (toe-pinch) were transcardially perfused with PBS and 4% PFA in PBS, pH 7.2. After perfusion, brains were removed and kept in 4% PFA in PBS, pH 7.2 at 4 °C for 5 h for post fixation. Seventy µm thick sagittal sections of brains were sectioned with a Leica VT1000S vibratome. Free floating brain sections were blocked overnight at 4 °C using blocking solution (1% BSA, 0.1% Triton X-100 in PBS). Primary antibodies were incubated for two days at 4 °C. Before adding secondary antibodies, sections were washed 3 × 10 min with 0.5% BSA, 0.05% Triton X-100 in PBS. The following primary antibodies were used: rabbit anti-mouse collagen-IV (Bio-Rad, 2150–1470, 1:300), goat anti-mouse CD13 (R&D Systems, AF2335, 1:100), mouse anti-human ASMA-FITC (Sigma, F37777, 1:100), goat anti-mouse podocalyxin (R&D Systems, AF1556, 1:100), rabbit anti-mouse Slc16a1 (Origene, TA321556, 1:100), rabbit anti-mouse Vwf (DAKO, A0082, 1:100),. Secondary antibodies were incubated overnight at 4 °C, followed by a wash 3 × 10 min in 0.5% BSA, 0.05% Triton X-100 in PBS. All fluorescently labeled secondary antibodies suitable for multiple labeling (donkey anti-goat IgG (H + L) Cy3 (#705-165-147), donkey anti-goat IgG (H + L) AlexaFluor647 (#705–605-147), donkey anti-rabbit IgG (H + L) AlexaFluor488 (#711-545-152), donkey anti-rabbit IgG (H + L) Cy3 (#711-165-152) were purchased from Jackson Immunoresearch. After 7 min incubation in a 1:10,000 4′,6-diamidino-2-phenylindole (DAPI) (Sigma, 10236276001) solution, sections were washed with PBS and mounted in Prolong Gold Antifade Reagent (Invitrogen, P36930). Sections were imaged using a confocal microscope (Leica SP5, objective 20 × and numerical aperture 0.7). Imaris software (Bitplane) and ImageJ were used for image processing and analysis. Salt-and-pepper noise was removed using a median filter with a radius of 1 pixel (ImageJ) or a median filter of 3 × 3x1 (Imaris).

### Quantification of vessel density and pericyte coverage

Sagittal Sections (70 µm thick) were stained with antibodies against collagen IV (COL-IV) and aminopeptidase N (CD13). Images of 22 µm z-size were analyzed using FIJI version 11 [60]. For vessel density, a slightly modified vessel analysis plugin (version 1.1) in FIJI was used, where 3 regions of interest (ROI) were measured in images taken from hippocampus or from cortex. To determine vessel pericyte coverage, images were converted to binary and 6 (150 μm × 150 µm) regions of interest were analyzed in COL-IV and CD13 channel using the area measurement tool. The percentage of signal overlay was calculated to determine the vessel pericyte coverage.

### Isolation of brain endothelial cells using flow cytometry

Two months old mice were deeply anesthetized using 25% Ketamine (50 mg/mL), 10% Xylazine (20 mg/mL) and after a loss of response to reflex stimulation (toe-pinch) the brain was removed. Hippocampus and cortex were dissected, coarsely mechanically dissociated using scissors, followed by enzymatical dissociation with 500 µl per brain region of a dissociation solution containing 1.55 g/L glucose, 0.08 W U/ml Liberase DH (Sigma, 5401054001), 100 U/ml DNaseI (Sigma, D4263-5VL) in HBSS (Gibco, 14065-049), at 37 °C for 20 min. After 10 min, tissue was gently mixed using a glass Pasteur pipette. After enzymatic dissociation, tissue was gently pushed 20 times through a 20G needle. For size exclusion, cells were passed through a 40 µm mesh and washed with 10 ml PBS. After washing and pelleting (300 g, 10 min, 4 °C), cells were prepared for myelin removal using myelin removal beads (130–096-731) and LS columns (130-042-401) according to the manufacturer`s protocol (Miltenyi Biotec). After myelin removal, cells were washed with FACS buffer (2% FBS, 0.01% NaN_3_ in PBS) and subsequently stained with directly conjugated antibodies for 30 min in 100 µl antibody solution. The following antibodies were used: anti-mouse CD45-PE (BD Bioscience, 553081, 1:20), anti-mouse CD11b-APC-Cy7 (BD Biosciences, 557657, 1:20) and anti-mouse CD31-APC (BD Biosciences, 551262, 1:20). Cells were washed with FACS buffer and kept in 200 µl FACS buffer until analysis. Live and dead staining using 7AAD-PerCP-Cy5.5 (559925, BD Biosciences) was performed directly before sorting. All procedures after tissue dissociation were performed on ice or 4 °C. OneComp eBeads™ (ThermoFischer Scientific, 01.1111.42) diluted in PBS were used for compensation. For each compensation, 0.5 µl antibody was added to the compensation beads. Live and dead staining was compensated by using a small fraction of stained sample for live and dead staining only.

Sorting of endothelial cells was performed using an ARIA III 5L cell sorter (BD) with 85 µm nozzle size, 1000 events/s with a flow rate of 1. Gating analyses included single cell gating, live cell gating (7AAD), negative gating for immune cells (CD45^+^, CD11b^+^) and positive gating for endothelial cells (CD31^+^). Cells were sorted using a 4-way purity mask directly into the RLT lysis buffer (Qiagen AllPrep Mini Kit, 80004) with β-mercaptoethanol (Sigma, M6250). After sorting, total RNA was isolated using the RNeasy Micro-Kit (Qiagen AllPrep Mini Kit, 80004). RNA quality was checked using an RNA Nano-Kit for BioAnalyzer 6000 (Agilent).

### RNA sequencing

RNA sequencing of samples was performed at the Functional Genomics Center (FGCZ) at the University of Zürich and ETH. Total RNA was depleted from rRNA and the library was prepared using the SMARTer Stranded Total RNA-seq Pico Input Mammalian Kit (Cat. 634839, Clontech Laboratories). Samples were sequenced with an Illumina HiSeq4000, single end, with a read length of 125 bp. Sequencing depth was 30–50 Mio reads.

### Bioinformatics analysis of RNA seq data

Raw sequencing read alignment and gene count summarization were performed using the bcbio-nextgen pipeline (v.19.03, https://github.com/bcbio/bcbio-nextgen). Quality encoding of sequencing reads was converted to Sanger format using seqkt (https://github.com/lh3/seqtk). Sequencing reads were aligned to the *Mus musculus* genome (mm10) using HISAT2 (v.2.1.0, [61]). Sequence alignment map files were converted to binary alignment map files and indexed, and alignment statistics were determined using samtools (v.1.9, [62]). Read count summarization at the gene level was performed using the featureCounts method of the Subread package (v.1.4.4, [63]) using the mm10 genome transcriptome annotation (v. 2018-10-10_92). Gene body coverage estimation was performed using the RSeQC package (v.4.0.0, [64]).

Analysis of the pre-processed raw counts was performed using R packages included in the online web application iDEP.92 [29]. First, raw counts were converted to log_2_ (counts per million (CPM) + 1) using the edgeR package. Genes were filtered to remove the ones expressed at less than 0.5 cpm (counts per million) in at least 1 sample. A principal component analysis (PCA) was performed using the log_2_(cpm + 1) data of all retained genes. Genes were centered and the heatmap.2 function of the gplots package was used to generate a heatmap using the 1000 most variable genes. Distance metrics for the sample and gene dendrograms were based on 1-Pearson’s correlation coefficient, followed by hierarchical clustering using the average linkage procedure. Differential gene expression analysis was performed using DESeq2 [65]. Testing for differential gene expression between different groups (male vs female, cortex vs hippocampus, long inbred vs recently wild-derived) was performed separately with a Benjamini–Hochberg FDR corrected value cutoff of 0.05. For each pairwise comparison, over-representation analysis of Gene Ontology (GO) biological process gene sets was performed for up-regulated and down-regulated genes separately, using hypergeometric tests followed by FDR value adjustment. Expression values of cell type marker genes were calculated as follows: Raw counts were normalized and converted to log_2_ using DESeq2. Average expression levels of cell type marker genes (22) were calculated and visualized with boxplots. RNA sequencing data, both raw data and gene-by-sample matrix of raw counts, were deposited in Gene Expression Omnibus (GEO) under accession number GSE173793.

## Supplementary Information

**Additional file 1: Figure S1.** Vessel density, pericyte coverage and BBB permeability in aged mice of different strains. Mean lifespan in days of A/J, C57BL/6J, DBA/2J strains. Data are from the YUAN 2 median lifespan dataset of the JAX phenome database (https://phenome.jax.org/measures/23201) (**A**).Vessel density (**B**) and pericyte coverage (**C**) in cortex and hippocampus of 2- month and 12- month-old mice. Mean permeability index to NaF of 2- and 12- month-old animals of A/J, C57BL/6J, DBA/2J strains in cortex (**D**) and hippocampus (**E**). Ratio of PI (hippocampus / cortex) of A/J, C57BL/6J, DBA/2J strains in 2- and 12-months animals (**F**). Pink dotted line: PI = 1. Each dot indicates one mouse. Both, female and male mice were used for analysis. Statistical analysis: unpaired two-tailed t-test or Mann Whitney test for pairwise comparison of 2- and 12- months old animals of one strain. * p-value < 0.05, ** p-value < 0.01. Data are presented as mean ± SD. **Figure S2.** Average expression levels of cell type marker genes in EC enriched samples. Average expression level (log_2_ normalized counts per million [CPM]) per sample of cell type marker genes for endothelial cells (**A**), arterial smooth muscle cells (**B**), pericytes (**C**), astrocytes (**D**), microglia (**E**), and vessel-associated fibroblasts 1 and -2 (**F**). **Figure S3.** Expression values per sample of selected DEG in females *vs* males. Expression levels of genes implicated in mitochondrial (**A**) or endothelial (**B**) function. Data are presented as mean ± SD. **Figure S4.** Expression values per sample for selected DEG in wild-derived *vs* long-inbred strains. Boxplots showing the expression per sample of DEG in recently wild-derived *vs* long-inbred strains. Gene expression of transporters expressed on brain EC (**A-B**). Expression of receptor-mediated transporters on brain EC (**C**). Expression of genes involved in vascular development and homeostasis (**D**, **E**). Gene expression levels of genes involved in immune response (**F**). Data are presented as mean ± SD.

**Additional file 2: Table S1.** Top 1000 deregulated genes used for unsupervised clustering of samples analyzed with RNA sequencing

**Additional file 3: Table S2.** DEG and enriched biological processes (BP) in endothelial cells isolated from female mice compared to endothelial cells isolated from male mice.

**Additional file 4: Table S3.** DEG and enriched biological processes in endothelial cells isolated from recently wild-derived mice compared to endothelial cells isolated from long-inbred strains.

## Abbreviations

ABC: ATP binding cassette, efflux transporter
ATP: Adenosine triphosphate
A-V zonation: Arteriovenous zonation
BBB: Blood–brain barrier
CNS: Central nervous system
CTX: Cortex
DEG: Differentially expressed gene
FDR: False discovery rate
EC: Endothelial cells
HC: Hippocampus
NaF: Sodium fluorescein
PI: Permeability index
SLC: Solute carrier, influx transporter
SNP: Single nucleotide polymorphism

## References

[1] Oakley R, Tharakan B (2014). Vascular hyperpermeability and aging. Aging Dis.

[2] Montagne A, Barnes SR, Sweeney MD, Halliday MR, Sagare AP, Zhao Z (2015). Blood-brain barrier breakdown in the aging human hippocampus. Neuron.

[3] Sohet F, Daneman R (2013). Genetic mouse models to study blood-brain barrier development and function. Fluids Barriers CNS.

[4] Sittig LJ, Carbonetto P, Engel KA, Krauss KS, Barrios-Camacho CM, Palmer AA (2016). Genetic background limits generalizability of genotype-phenotype relationships. Neuron.

[5] Weatherall DJ (2000). Single gene disorders or complex traits: lessons from the thalassaemias and other monogenic diseases. BMJ (Clinical research ed).

[6] Keane TM, Goodstadt L, Danecek P, White MA, Wong K, Yalcin B (2011). Mouse genomic variation and its effect on phenotypes and gene regulation. Nature.

[7] Ryan MJ, Didion SP, Davis DR, Faraci FM, Sigmund CD (2002). Endothelial dysfunction and blood pressure variability in selected inbred mouse strains. Arterioscler Thromb Vasc Biol.

[8] Kim SK, Avila JJ, Massett MP (2020). Interaction of genetic background and exercise training intensity on endothelial function in mouse aorta. Korean J Physiol Pharmacol.

[9] Kempermann G, Brandon EP, Gage FH (1998). Environmental stimulation of 129/SvJ mice causes increased cell proliferation and neurogenesis in the adult dentate gyrus. Curr Biol.

[10] Kempermann G, Gage FH (2002). Genetic influence on phenotypic differentiation in adult hippocampal neurogenesis. Brain Res Dev Brain Res.

[11] Kempermann G, Chesler EJ, Lu L, Williams RW, Gage FH (2006). Natural variation and genetic covariance in adult hippocampal neurogenesis. Proc Natl Acad Sci USA.

[12] Crawley JN. What's wrong with my mouse?: Behavioral phenotyping of transgenic and knockout mice; 2007.

[13] Lee HK, Widmayer SJ, Huang MN, Aylor DL, Marchuk DA (2019). Novel neuroprotective loci modulating ischemic stroke volume in wild-derived inbred mouse strains. Genetics.

[14] Omura T, Omura K, Tedeschi A, Riva P, Painter MW, Rojas L (2015). Robust axonal regeneration occurs in the injured CAST/Ei mouse CNS. Neuron.

[15] Qian B, Rudy RF, Cai T, Du R (2018). Cerebral artery diameter in inbred mice varies as a function of strain. Front Neuroanat.

[16] Onos KD, Uyar A, Keezer KJ, Jackson HM, Preuss C, Acklin CJ (2019). Enhancing face validity of mouse models of Alzheimer's disease with natural genetic variation. PLoS Genet.

[17] Silver LM (1995). Mouse genetics: concepts and applications.

[18] Beck JA, Lloyd S, Hafezparast M, Lennon-Pierce M, Eppig JT, Festing MF (2000). Genealogies of mouse inbred strains. Nat Genet.

[19] Daneman R, Zhou L, Kebede AA, Barres BA (2010). Pericytes are required for blood-brain barrier integrity during embryogenesis. Nature.

[20] Armulik A, Genové G, Mäe M, Nisancioglu MH, Wallgard E, Niaudet C (2010). Pericytes regulate the blood-brain barrier. Nature.

[21] Mäe MA, He L, Nordling S, Vazquez-Liebanas E, Nahar K, Jung B (2021). Single-cell analysis of blood-brain barrier response to pericyte loss. Circ Res.

[22] Vanlandewijck M, He L, Mäe MA, Andrae J, Ando K, Del Gaudio F (2018). A molecular atlas of cell types and zonation in the brain vasculature. Nature.

[23] Saunders NR, Dziegielewska KM, Møllgård K, Habgood MD (2015). Markers for blood-brain barrier integrity: how appropriate is Evans blue in the twenty-first century and what are the alternatives?. Front Neurosci.

[24] Devraj K, Guérit S, Macas J, Reiss Y (2018). An in vivo blood-brain barrier permeability assay in mice us. J Vis Exp.

[25] Tavazoie M, Van der Veken L, Silva-Vargas V, Louissaint M, Colonna L, Zaidi B (2008). A specialized vascular niche for adult neural stem cells. Cell Stem Cell.

[26] Yuan R, Meng Q, Nautiyal J, Flurkey K, Tsaih SW, Krier R (2012). Genetic coregulation of age of female sexual maturation and lifespan through circulating IGF1 among inbred mouse strains. Proc Natl Acad Sci USA.

[27] Yang AC, Stevens MY, Chen MB, Lee DP, Stähli D, Gate D (2020). Physiological blood-brain transport is impaired with age by a shift in transcytosis. Nature.

[28] Obermeier B, Daneman R, Ransohoff RM (2013). Development, maintenance and disruption of the blood-brain barrier. Nat Med.

[29] Ge SX, Son EW, Yao R (2018). iDEP: an integrated web application for differential expression and pathway analysis of RNA-Seq data. BMC Bioinformatics.

[30] Paik DT, Tian L, Williams IM, Rhee S, Zhang H, Liu C (2020). Single-cell RNA-seq unveils unique transcriptomic signatures of organ-specific endothelial cells. Circulation.

[31] Poltorak A, Apalko S, Sherbak S (2018). Wild-derived mice: from genetic diversity to variation in immune responses. Mamm Genome.

[32] Leist SR, Pilzner C, van den Brand JM, Dengler L, Geffers R, Kuiken T (2016). Influenza H3N2 infection of the collaborative cross founder strains reveals highly divergent host responses and identifies a unique phenotype in CAST/EiJ mice. BMC Genomics.

[33] Todorov MI, Paetzold JC, Schoppe O, Tetteh G, Shit S, Efremov V (2020). Machine learning analysis of whole mouse brain vasculature. Nat Methods.

[34] Li Y, Choi WJ, Wei W, Song S, Zhang Q, Liu J (2018). Aging-associated changes in cerebral vasculature and blood flow as determined by quantitative optical coherence tomography angiography. Neurobiol Aging.

[35] Chen J, Sivan U, Tan SL, Lippo L, De Angelis J, Labella R (2021). High-resolution 3D imaging uncovers organ-specific vascular control of tissue aging. Sci Adv.

[36] Yang S, Gu C, Mandeville ET, Dong Y, Esposito E, Zhang Y (2017). Anesthesia and surgery impair blood-brain barrier and cognitive function in mice. Front Immunol.

[37] Kutuzov N, Flyvbjerg H, Lauritzen M (2018). Contributions of the glycocalyx, endothelium, and extravascular compartment to the blood-brain barrier. Proc Natl Acad Sci USA.

[38] Patik I, Kovacsics D, Német O, Gera M, Várady G, Stieger B (2015). Functional expression of the 11 human Organic Anion Transporting Polypeptides in insect cells reveals that sodium fluorescein is a general OATP substrate. Biochem Pharmacol.

[39] Sun H, Miller DW, Elmquist WF (2001). Effect of probenecid on fluorescein transport in the central nervous system using in vitro and in vivo models. Pharm Res.

[40] Gao B, Vavricka SR, Meier PJ, Stieger B (2015). Differential cellular expression of organic anion transporting peptides OATP1A2 and OATP2B1 in the human retina and brain: implications for carrier-mediated transport of neuropeptides and neurosteriods in the CNS. Pflugers Arch.

[41] Hawkins BT, Ocheltree SM, Norwood KM, Egleton RD (2007). Decreased blood-brain barrier permeability to fluorescein in streptozotocin-treated rats. Neurosci Lett.

[42] Soontornmalai A, Vlaming ML, Fritschy JM (2006). Differential, strain-specific cellular and subcellular distribution of multidrug transporters in murine choroid plexus and blood-brain barrier. Neuroscience.

[43] Farhat F, Amérand A, Simon B, Guegueniat N, Moisan C (2017). Gender-dependent differences of mitochondrial function and oxidative stress in rat skeletal muscle at rest and after exercise training. Redox Rep.

[44] Sultanova RF, Schibalski R, Yankelevich IA, Stadler K, Ilatovskaya DV (2020). Sex differences in renal mitochondrial function: a hormone-gous opportunity for research. Am J Physiol Renal Physiol.

[45] Silaidos C, Pilatus U, Grewal R, Matura S, Lienerth B, Pantel J (2018). Sex-associated differences in mitochondrial function in human peripheral blood mononuclear cells (PBMCs) and brain. Biol Sex Differ.

[46] Norheim F, Hasin-Brumshtein Y, Vergnes L, Chella Krishnan K, Pan C, Seldin MM (2019). Gene-by-sex interactions in mitochondrial functions and cardio-metabolic traits. Cell Metab.

[47] Huang X, Shen W, Veizades S, Liang G, Sayed N, Nguyen PK (2021). Single-cell transcriptional profiling reveals sex and age diversity of gene expression in mouse endothelial cells. Front Genet.

[48] Pierce SB, Gersak K, Michaelson-Cohen R, Walsh T, Lee MK, Malach D (2013). Mutations in LARS2, encoding mitochondrial leucyl-tRNA synthetase, lead to premature ovarian failure and hearing loss in Perrault syndrome. Am J Hum Genet.

[49] Cikic S, Chandra PK, Harman JC, Rutkai I, Katakam PV, Guidry JJ (2021). Sexual differences in mitochondrial and related proteins in rat cerebral microvessels: a proteomic approach. J Cerebral Blood Flow Metab.

[50] Shavit JA, Manichaikul A, Lemmerhirt HL, Broman KW, Ginsburg D (2009). Modifiers of von Willebrand factor identified by natural variation in inbred strains of mice. Blood.

[51] McCandless EE, Wang Q, Woerner BM, Harper JM, Klein RS (2006). CXCL12 limits inflammation by localizing mononuclear infiltrates to the perivascular space during experimental autoimmune encephalomyelitis. J Immunol.

[52] Augustin HG, Koh GY, Thurston G, Alitalo K (2009). Control of vascular morphogenesis and homeostasis through the angiopoietin-Tie system. Nat Rev Mol Cell Biol.

[53] Segarra M, Aburto MR, Cop F, Llao-Cid C, Hartl R, Damm M (2018). Endothelial Dab1 signaling orchestrates neuro-glia-vessel communication in the central nervous system. Science.

[54] Shen GQ, Girelli D, Li L, Rao S, Archacki S, Olivieri O (2014). A novel molecular diagnostic marker for familial and early-onset coronary artery disease and myocardial infarction in the LRP8 gene. Circ Cardiovasc Genet.

[55] Ulrich V, Konaniah ES, Herz J, Gerard RD, Jung E, Yuhanna IS (2014). Genetic variants of ApoE and ApoER2 differentially modulate endothelial function. Proc Natl Acad Sci USA.

[56] Berry DC, Jacobs H, Marwarha G, Gely-Pernot A, O'Byrne SM, DeSantis D (2013). The STRA6 receptor is essential for retinol-binding protein-induced insulin resistance but not for maintaining vitamin A homeostasis in tissues other than the eye. J Biol Chem.

[57] Farjo KM, Farjo RA, Halsey S, Moiseyev G, Ma JX (2012). Retinol-binding protein 4 induces inflammation in human endothelial cells by an NADPH oxidase- and nuclear factor kappa B-dependent and retinol-independent mechanism. Mol Cell Biol.

[58] Xiao Q, Zhou Y, Lauschke VM (2020). Ethnogeographic and inter-individual variability of human ABC transporters. Hum Genet.

[59] Wahlsten D, Metten P, Crabbe JC (2003). A rating scale for wildness and ease of handling laboratory mice: results for 21 inbred strains tested in two laboratories. Genes Brain Behav.

[60] Schindelin J, Arganda-Carreras I, Frise E, Kaynig V, Longair M, Pietzsch T (2012). Fiji: an open-source platform for biological-image analysis. Nat Methods.

[61] Kim D, Paggi JM, Park C, Bennett C, Salzberg SL (2019). Graph-based genome alignment and genotyping with HISAT2 and HISAT-genotype. Nat Biotechnol.

[62] Li H, Handsaker B, Wysoker A, Fennell T, Ruan J, Homer N (2009). The Sequence Alignment/Map format and SAMtools. Bioinformatics (Oxford, England).

[63] Liao Y, Smyth GK, Shi W (2014). featureCounts: an efficient general purpose program for assigning sequence reads to genomic features. Bioinformatics (Oxford, England).

[64] Wang L, Wang S, Li W (2012). RSeQC: quality control of RNA-seq experiments. Bioinformatics (Oxford, England).

[65] Love MI, Huber W, Anders S (2014). Moderated estimation of fold change and dispersion for RNA-seq data with DESeq2. Genome Biol.

