# Supplementary figures and images for: Characterization of the blood–brain barrier in genetically diverse laboratory mouse strains
Source: Fluids Barriers CNS. 2021 Jul 28;18:34. doi: 10.1186/s12987-021-00269-w (PMC8317333; doi:10.1186/s12987-021-00269-w)

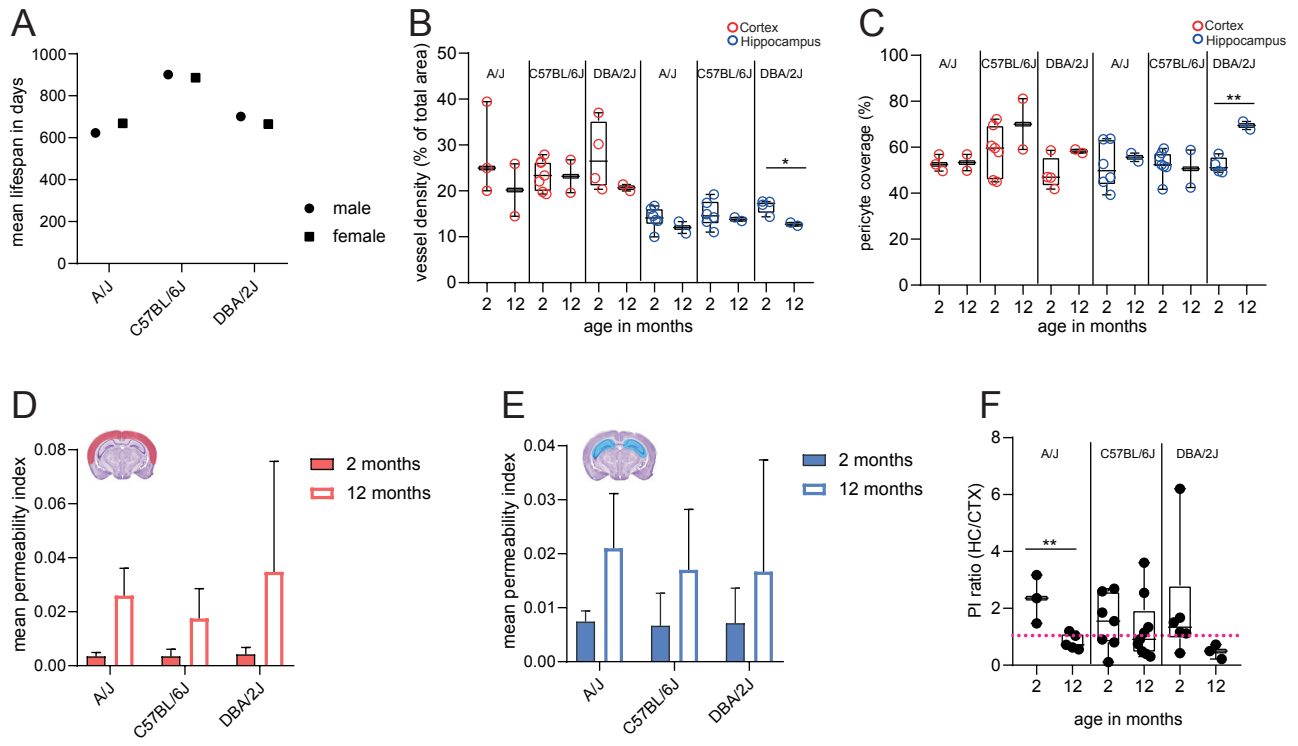

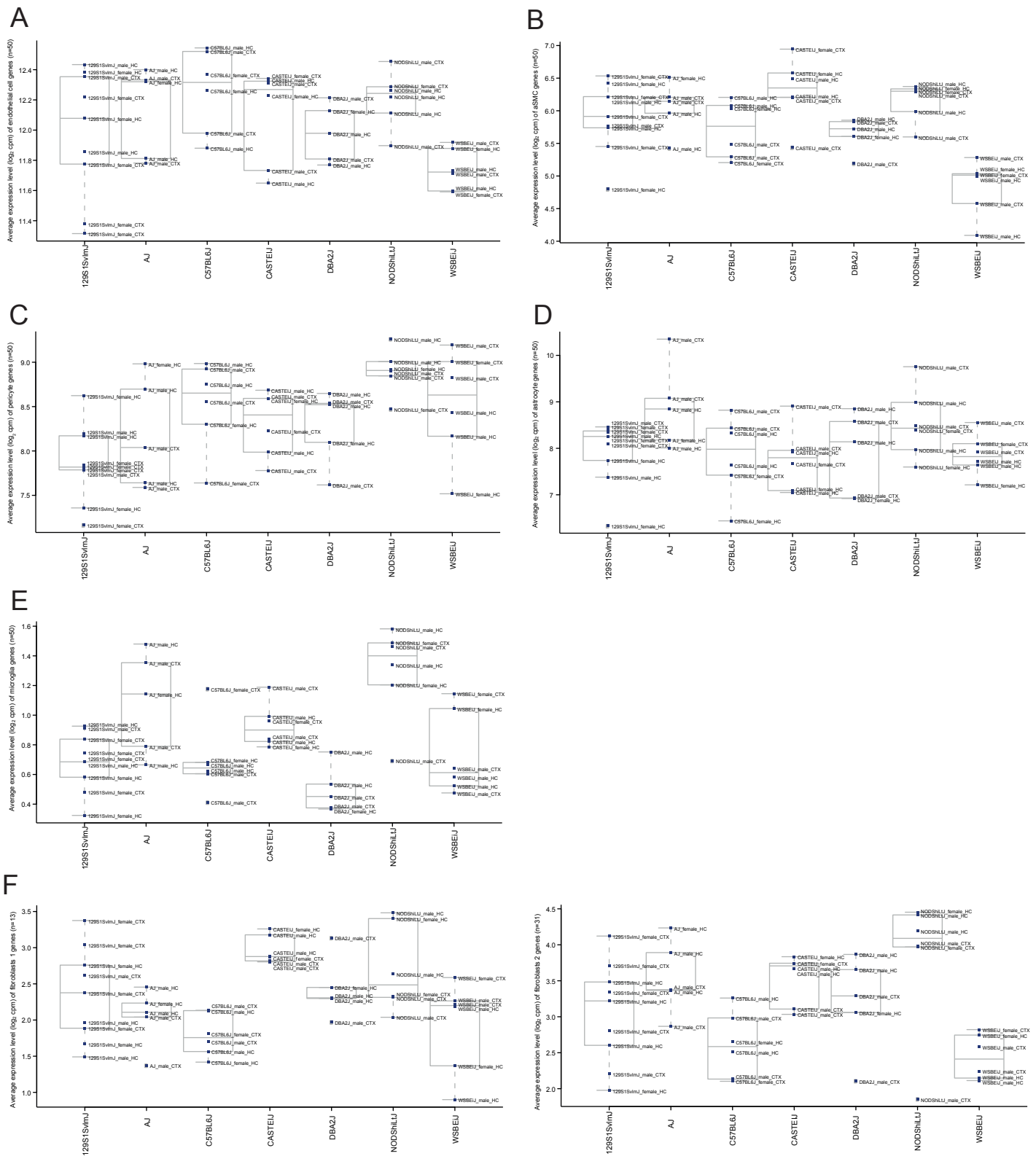

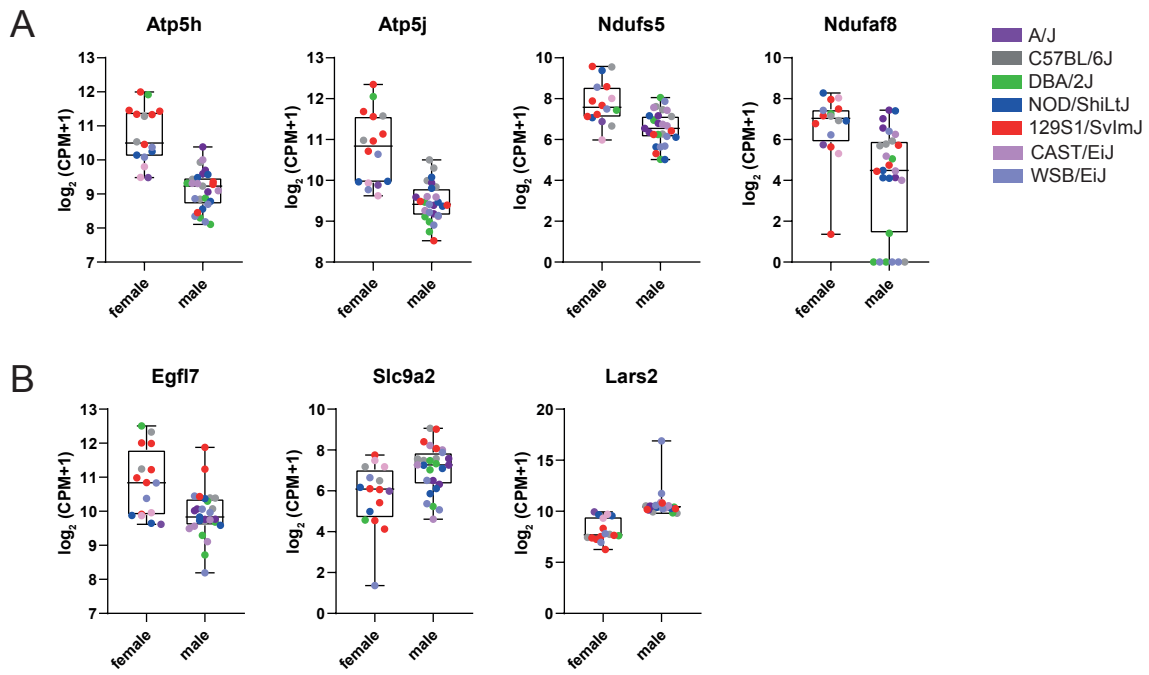

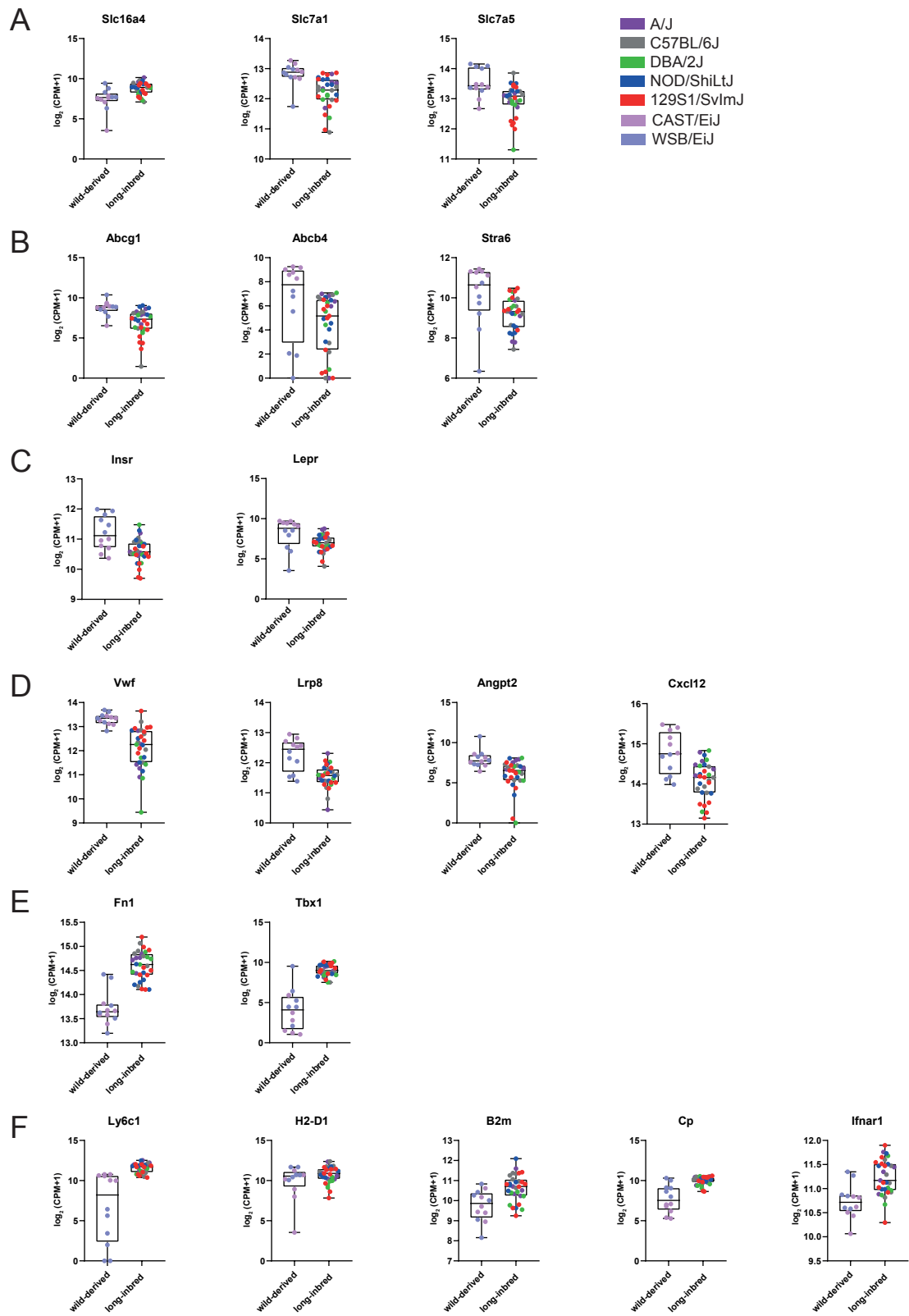

Supplement: Supplementary file 1 — Additional file 1: Figure S1. Vessel density, pericyte coverage and BBB permeability in aged mice of different strains. Mean lifespan in days of A/J, C57BL/6J, DBA/2J strains. Data are from the YUAN 2 median lifespan dataset of the JAX phenome database (https://phenome.jax.org/measures/23201) (A).Vessel density (B) and pericyte coverage (C) in cortex and hippocampus of 2- month and 12- month-old mice. Mean permeability index to NaF of 2- and 12- month-old animals of A/J, C57BL/6J, DBA/2J strains in cortex (D) and hippocampus (E). Ratio of PI (hippocampus / cortex) of A/J, C57BL/6J, DBA/2J strains in 2- and 12-months animals (F). Pink dotted line: PI = 1. Each dot indicates one mouse. Both, female and male mice were used for analysis. Statistical analysis: unpaired two-tailed t-test or Mann Whitney test for pairwise comparison of 2- and 12- months old animals of one strain. * p-value < 0.05, ** p-value < 0.01. Data are presented as mean ± SD. Figure S2. Average expression levels of cell type marker genes in EC enriched samples. Average expression level (log2 normalized counts per million [CPM]) per sample of cell type marker genes for endothelial cells (A), arterial smooth muscle cells (B), pericytes (C), astrocytes (D), microglia (E), and vessel-associated fibroblasts 1 and -2 (F). Figure S3. Expression values per sample of selected DEG in females vs males. Expression levels of genes implicated in mitochondrial (A) or endothelial (B) function. Data are presented as mean ± SD. Figure S4. Expression values per sample for selected DEG in wild-derived vs long-inbred strains. Boxplots showing the expression per sample of DEG in recently wild-derived vs long-inbred strains. Gene expression of transporters expressed on brain EC (A-B). Expression of receptor-mediated transporters on brain EC (C). Expression of genes involved in vascular development and homeostasis (D, E). Gene expression levels of genes involved in immune response (F). Data are presented as mean ± SD. [file 12987_2021_269_MOESM1_ESM.pdf]
